# Fibrosing mediastinitis mimicking as chronic pulmonary thromboembolism

**DOI:** 10.1259/bjrcr.20190049

**Published:** 2020-02-12

**Authors:** Dimpi Sinha, Nischal G Kundaragi, Sudhir Kumar Kale, Sukrity Sharma

**Affiliations:** 1 Department of Radiology, Aster CMI Hospital, Bangalore, India; 2 Department of Interventinal Radiology, Aster CMI Hospital, Bangalore, India

## Abstract

Fibrosing mediastinitis is an uncommon, benign, progressive disorder caused by proliferation of fibrous tissue within mediastinum resulting in encasement of vital mediastinal broncho-vascular structures. Due to its rarity and variable clinical presentation, it is often misdiagnosed. We are presenting a case of fibrosing mediastinitis in an Ethiopian origin young male presenting with pulmonary hypertension due to simultaneous occlusion of pulmonary vein and arteries, clinically misdiagnosed as chronic pulmonary thromboembolism.

## Case summary

### Clinical presentation

A 31-year-old Ethiopian origin male presented with gradually progressive dyspnoea, dry cough with occasional streaky haemoptysis and weight loss for 2–3 years. There was no history of fever. Oxygen saturation at room air was ~80%. Ventilation perfusion scan performed outside showed virtually no perfusion in the right lung and hypo-perfusion in lower lobe of the left lung. Based on these findings, a clinical diagnosis of chronic thromboembolic pulmonary hypertension was made by local physicians and was treated for the same for more than 2 years without any significant clinical improvement. Thus, patient approached our centre for further management.

On clinical examination, patient was severely dyspnoeic with oxygen saturation at room air ~80%.

Echo-cardiography showed severe pulmonary hypertension with pulmonary artery systolic pressure of 110–120 mmHg, dilated right atrium and right ventricle with tricuspid regurgitation.

CT pulmonary angiography was performed to assess the status of presumed pulmonary artery thromboembolism.

### Imaging findings

**Figure 1.**
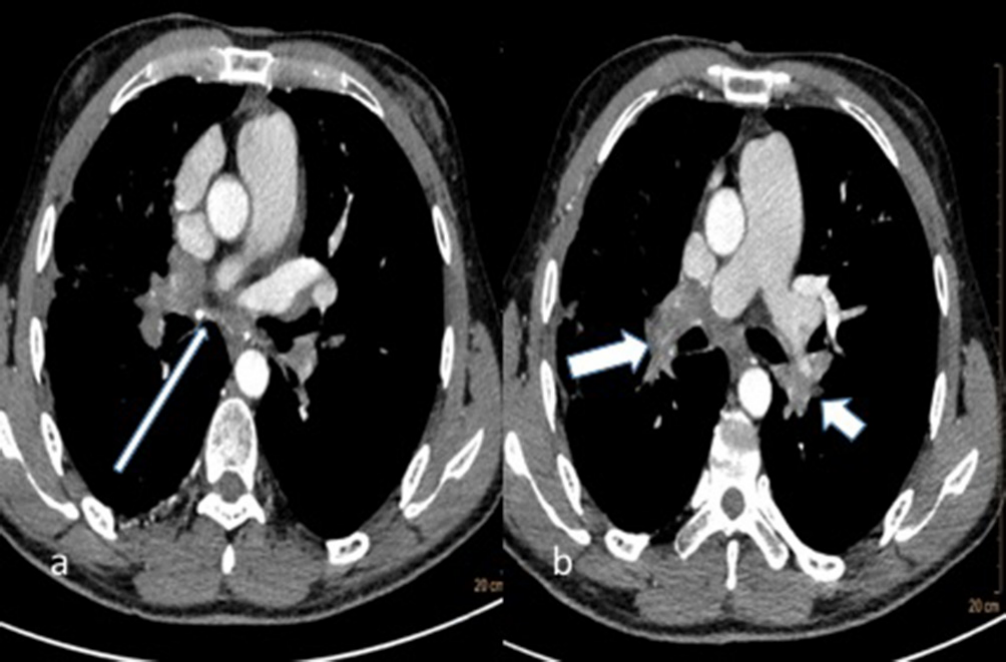
31-year-old male with fibrosing mediastinitis who presented with progressive dyspnoea and cough (a) axial CT chest section showing ill-defined middle mediastinal and (b) bilateral hilar soft tissue attenuation lesion (long and short thick white arrows) with specks of calcification (thin white arrow).

**Figure 2.**
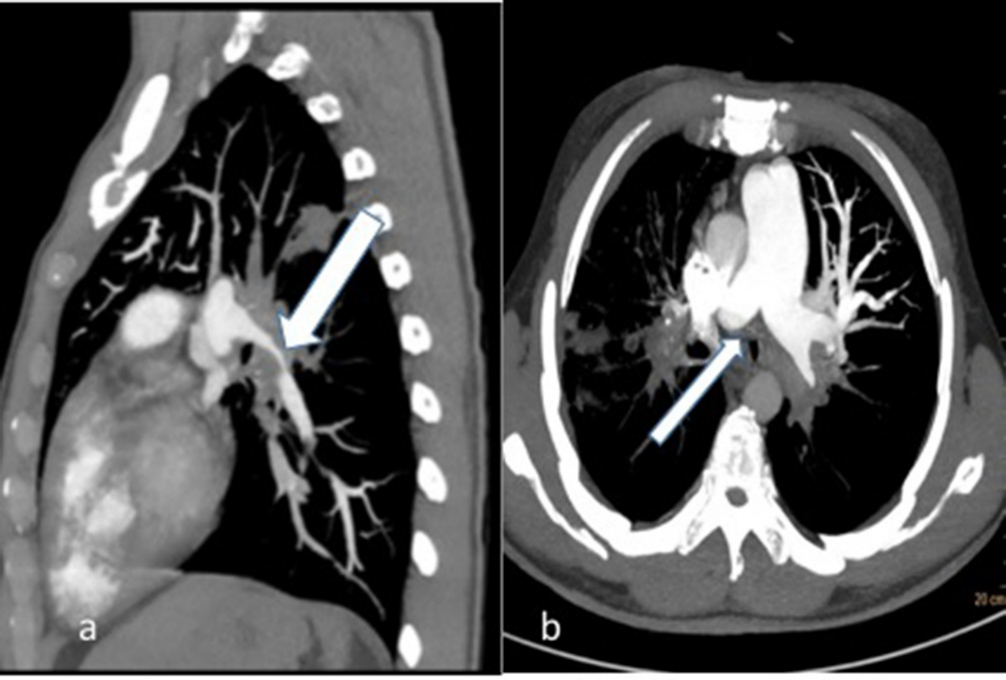
31-year-old male with fibrosing mediastinitis who presented with progressive dyspnoea and cough (a) 2D sagittal MIP reconstruction CT images showing left hilar soft tissue lesion causing short segment stenosis of the left lower lobar artery (thick white arrow) (b) axial 2D MIP reconstructed image showing main pulmonary artery dilatation and abrupt cut-off of right pulmonary artery (thin white arrow).

**Figure 3.**
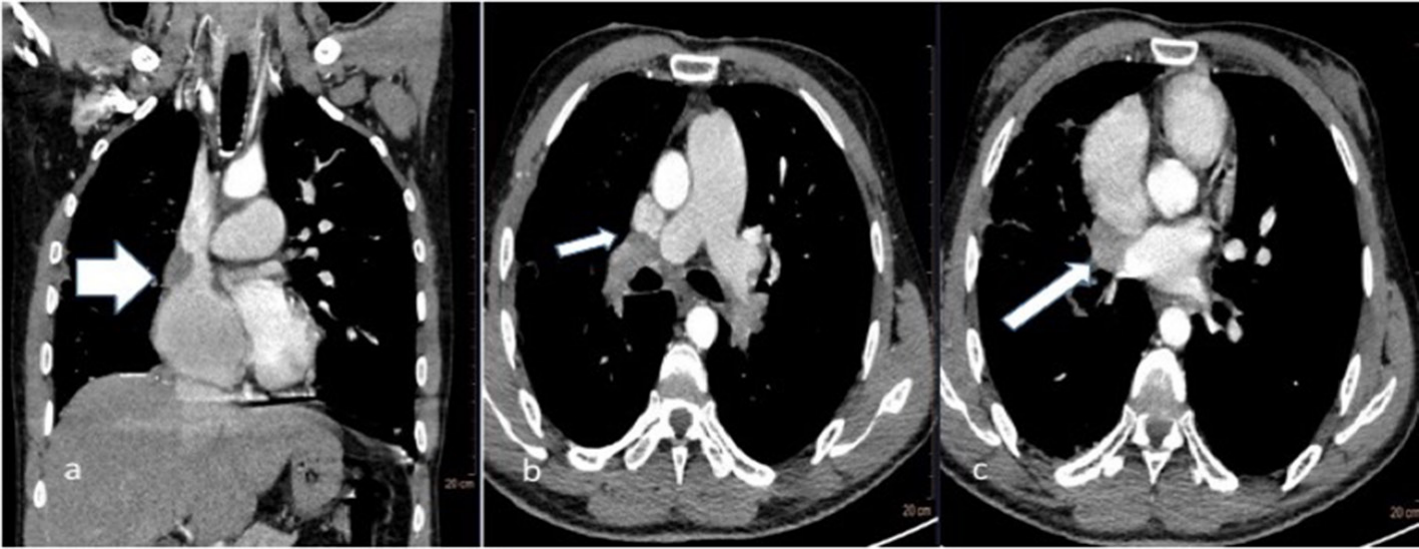
31-year-old male with fibrosing mediastinitis who presented with progressive dyspnoea and cough (a) coronal and (b) axial CT sections through chest showing indentation of Superior vena cava (SVC) by the soft tissue lesion at right hilum (short thick and thin white arrow).(c) axial CT section showing encasement of right superior pulmonary vein (long thin white arrow).

**Figure 4.**
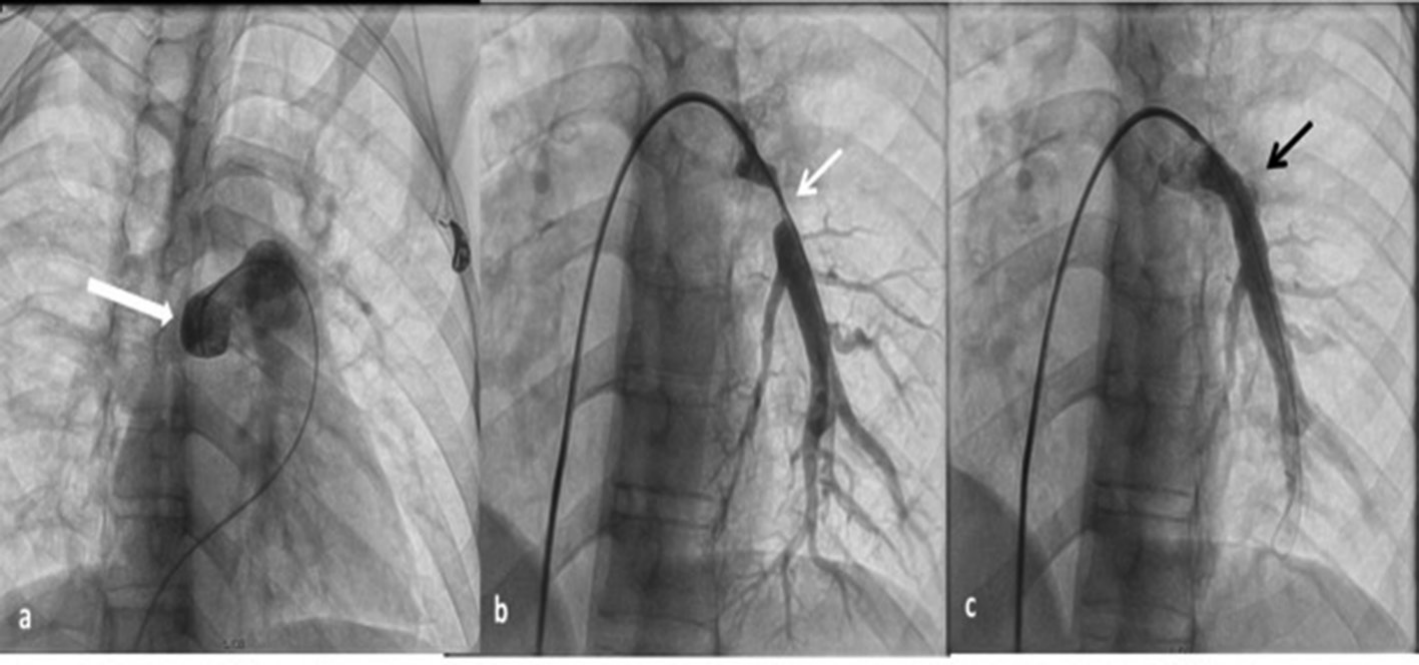
31-year-old male with fibrosing mediastinitis who presented with progressive dyspnoea and cough (a) digital subtraction angiography image showing cut-off of right pulmonary artery (thick white arrow) (b) short segment narrowing of left lower lobar pulmonary artery (thin white arrow). (c) poststenting image shows good flow across the stent (thin black arrow).

CT pulmonary angiogram revealed ill-defined, hypodense non-enhancing soft tissue density lesion with foci of calcification, involving bilateral hila (right >left) and middle mediastinum (Figure 1a, b). There was encasement of right pulmonary artery with complete luminal cut-off (Figure 2b). Complete non-opacification of right lobar and segmental pulmonary arteries was seen. The right superior pulmonary vein (Figure 3c) was encased with complete luminal occlusion. On the left side, severe short segment stenosis of the lower lobar pulmonary artery was seen (Figure 2a). There was posterior indentation on Superior vena cava (SVC) (3a and b). Bilateral lungs showed patchy areas of parenchymal consolidation and atelectatic bands. Digital subtraction angiography showed similar findings. (Figure 4a, b). Main and left branch pulmonary artery mean pressures were 64 mmHg and 62 mmHg, respectively. Mean pressures measured across the stenosis were as follows—prestenosis: 64 mmHg and poststenosis: 6 mmHg—and gradient was 58 mmHg.

### Management

Subsequently, the patient underwent bronchoscopy and trans-bronchial needle aspiration biopsy. Histopathologic examination showed non-specific changes with the presence of fibrosis and acellular collagen. There was no evidence of malignancy, fungal infection or granulomatous inflammation. Bronchio-alveolar lavage and GeneXpert were positive for acid fast bacillus.

Considering clinical, imaging and histopathology findings and after ruling out other possible diseases, a diagnosis of mediastinal fibrosis possibly secondary to tuberculosis was made.

**Figure 5.**
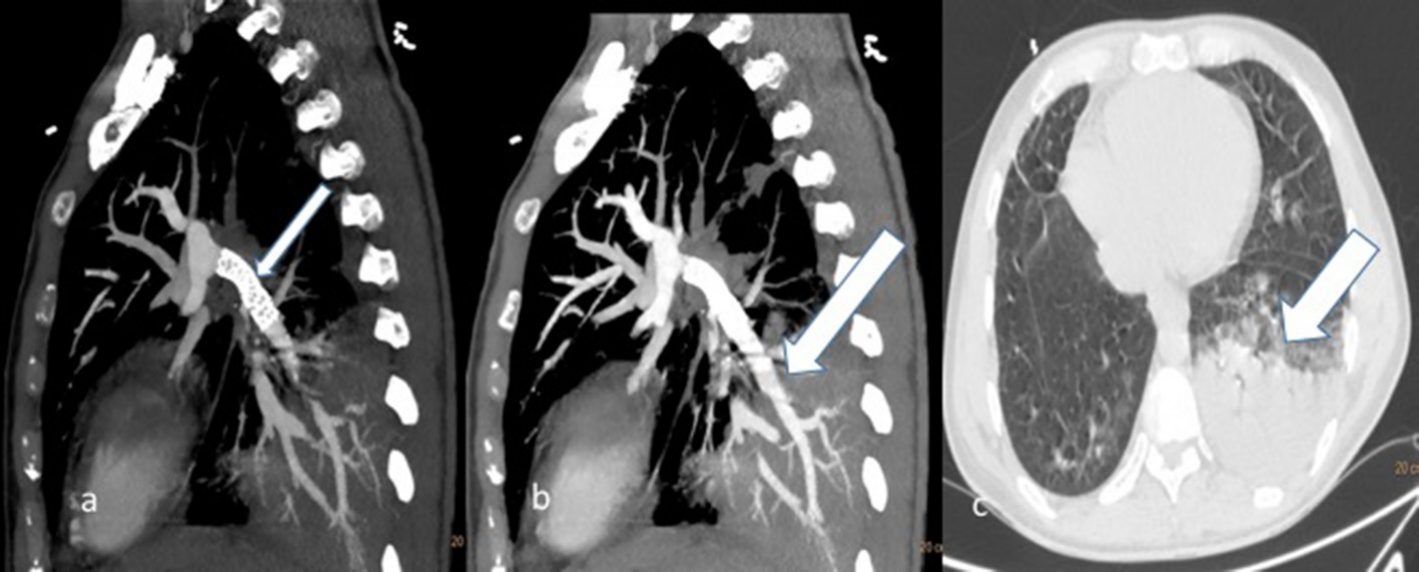
31-year-old male with fibrosing mediastinitis who presented with progressive dyspnoea and cough (a) and (b) postprocedure sagittal MIP reconstruction CT images showing endovascular stent in the left lower lobar pulmonary artery (thin long white arrow) with increased distal perfusion (long thick white arrow) (c) axial CT section lung window showing pulmonary haemorrhage in lower lobe of left lung (short thick white arrow).

The patient was started on antitubercular treatment. In view of the progressive breathlessness and severe stenosis of the lower lobar branch of left pulmonary artery, endovascular stenting under fluoroscopic guidance was done for symptomatic relief (Figures 4c and 5a and b). The right pulmonary artery recanalisation was not attempted because of chronic total occlusion and risk of arterial rupture. A 10 × 36 sized VALEO^®^ Vascular stent (Bard Inc. New Providence, NJ, USA) was placed across the stenosis. Poststenting main pulmonary artery mean pressure reduced to 48 from 64 mmHg and left lower lobe branch artery pressure increased from 6 to 38 mmHg. Gradient across stenosis significantly reduced from 58 to 10 mmHg. Procedure was uneventful.

10 h later, patient developed breathlessness and desaturation and was diagnosed with type two respiratory failure (alveolar hypoventilation leading to hypoxia and hypercapnia). He underwent a CT pulmonary angiography which revealed dense segmental pulmonary haemorrhage into left lower lobe likely representing reperfusion injury (Figure 5c). He was placed on Non Invasive Ventilation support, high flow O2, intravenous diuretics, intravenous antibiotics, intravenous anti fibrinolytics. Patient maintained a saturation between 85–90% on 100% FiO2. Despite aggressive medical management, patient succumbed to multi organ failure on day three.

## Discussion

Fibrosing mediastinitis is an uncommon benign disorder characterised by proliferation of dense fibrous tissue within the mediastinum.^1^ The condition leads to encasement, narrowing and sometimes complete occlusion, as was seen in our case, of vital vascular and non-vascular mediastinal structures.

Although it can present at any age, it typically presents in young adults.^2^ Mostly they are idiopathic.^3^

Two distinct patterns of fibrosing mediastinitis has been described in literature—focal form, affecting almost 82% of patients, most commonly affecting the right para-tracheal, subcarinal and hilar region. This pattern is frequently associated with calcifications and frequently consequential to histoplasmosis or less commonly tuberculosis.^1,2,4^The less common diffuse form (18 %) manifests as multicompartmental diffuse mediastinal infiltrating lesion, often non-calcified and are usually associated with autoimmune syndromes like retroperitoneal fibrosis, orbital pseudotumors, trauma, Hodgkin disease, and drug therapy with methysergide maleate.^2,5–8^

The precise aetiology and pathogenesis of fibrosing mediastinitis are unknown. According to several clinical and radiologic –pathologic series, granulomatous/focal fibrosing mediastinitis usually occur as a result of immune-mediated hypersensitivity response to histoplasma capsulatum or tubercular infection.^1,4^The idiopathic diffuse form of fibrosing mediastinitis is thought to be part of IgG4-RD spectrum, which includes conditions like retroperitoneal fibrosis or Riedel’s thyroiditis. Histologically, it is characterised by pauci-cellular collagen and fibrous tissue proliferation in mediastinal fat. It may contain infiltrates of mononuclear cells with eosinophilic cytoplasm.

Clinical presentation of fibrosing mediastinitis is variable based on the mediastinal structure involved. Compression or occlusion of SVC, pulmonary artery, pulmonary vein, central airways or oesophagus with resultant clinical syndromes have been described in literature. The most common presenting complaints include cough, dyspnoea, recurrent pulmonary infection, haemoptysis and pleuritic chest pain. Systemic symptoms such as fever and weight loss may be present.

SVC syndrome is the most common manifestation of fibrosing mediastinitis.^1,9^Obstruction of the central airways is fairly common in patients with fibrosing mediastinitis and typically manifests with cough and dyspnoea. Patients with pulmonary venous occlusion can present with progressive or exertional dyspnoea as well as with haemoptysis. Long-standing pulmonary venous occlusion can also result in secondary pulmonary arterial hypertension, cor pulmonale and pulmonary infarction.^10^Pulmonary arterial stenosis or occlusion results in pulmonary hypertension and is relatively an uncommon manifestation of fibrosing mediastinitis.

Diagnosis of mediastinal fibrosis is usually made on cross-section imaging. Although MRI has superior contrast resolution, it has lower sensitivity for picking up calcifications. CT is the most preferred modality for diagnostic evaluation of suspected or known case of fibrosing mediastinitis. On CT, fibrosing mediastinitis typically presents with a focal or diffuse infiltrating soft tissue attenuation lesion with or without calcifications, most commonly affecting middle mediastinum causing encasement of vital mediastinal structures. The anterior and posterior mediastinum are less commonly involved. Maximum intensity projection reconstructions accurately depict the extent, level, and length of stenosis of the involved vascular structure. In addition, it demonstrates the lung parenchymal changes such as infarcts due to venous/arterial involvement. Lympho-proliferative disorders and sarcoidosis are its main differential diagnosis on imaging.

Our case highlights the importance of cross-sectional imaging in reaching unto the definitive cause of pulmonary artery hypertension. In a young patient from endemic zone, presenting with progressive pulmonary hypertension, fibrosing mediastinitis should be considered in differential diagnosis along with other common causes like chronic pulmonary thromboembolism and primary pulmonary hypertension.

There is no definite treatment for fibrosing mediastinitis. Current treatment strategies aim at either halting disease progression by the use of immunosuppressant or at symptomatic relief by surgical or minimally invasive procedures.

Systemic immunosuppressant and corticosteroids are found to be beneficial in diffuse form of the disease as described in few case reports.^11^ They are of limited significance in granulomatous form of fibrosing mediastinitis.

Surgical approach is reserved for localised disease. Bilateral mediastinal involvement is a contraindication for surgery. Overall surgical therapy has high morbidity and mortality.^1^

Localised therapy is directed towards reopening of the occluded structure for symptom relief. This include endovascular, endo-bronchial or trans-oesophageal balloon dilatation/stenting.^12–15^In view of bilateral hilar involvement, endovascular stenting of stenosed pulmonary artery was considered as the best option in our case. Unfortunately, the patient died due to reperfusion injury.

Reperfusion injury is a delayed complication of pulmonary arterial stenting, usually develops 24–72 h after the procedure.^16,17^ Recent studies have reported an incidence of 7%.^18^ Earlier believed to be due to elevation of perfusion pressure in the poststenotic territory following stenting, the recent data suggests a role of microtrauma inflicted by the guide wire or the balloon leading to release of inflammatory cytokines with vascular oedema.^19^ Its incidence is more in high-grade vascular stenosis with severe haemodynamic compromise.

## Learning objectives

- Fibrosing mediastinitis is an uncommon progressive disease with grave prognosis resulting in extrinsic compression and occlusion of vital broncho-vascular mediastinal structures.
- Although pulmonary artery and venous stenosis with resultant pulmonary hypertension are rare manifestations of the disease, a high index of suspicion in young patients from endemic zone presenting with progressive dyspnoea, cough and haemoptysis could lead to correct diagnosis.
- Cross-sectional imaging particularly, contrast-enhanced CT, plays a vital role in making a diagnosis and assessing the severity of fibrosing mediastinitis.
